# Influence of Different Power Outputs of Er:YAG Laser on Shear Bond Strength of a Resin Composite to Feldspathic Porcelain

**Published:** 2015-03

**Authors:** Mostafa Sadeghi, Abdolrahim Davari, Amin Abolghasami Mahani, Hamid Hakimi

**Affiliations:** a Dept. of Operative Dentistry, School of Dentistry, Rafsanjan University of Medical Sciences, Rafsanjan, Iran.; b Dept. of Operative Dentistry, Member of Social Determinants of Oral Health Research Center, Dental School, Shahid Sadoughi University of Medical Sciences, Yazd, Iran.; c Postgraduate Student, Polymer Faculty, Tehran University, Tehran, Iran.; d Dept. of Microbiology, School of Medicine, Rafsanjan University of Medical Sciences, Rafsanjan, Iran.

**Keywords:** Porcelain repair, Er:YAG laser, Surface treatment, Shear bond strength, Resin composite, Hydrofluoric acid

## Abstract

**Statement of the Problem:**

Porcelain may fracture or chip if exposed to any traumas and can be repaired by using a resin composite.

**Purpose:**

This study was aimed to evaluate the influences of Er:YAG laser on shear bond strength (SBS) of resin composite to feldspathic porcelain.

**Materials and Method:**

Seventy-two porcelain blocks were divided into six groups (n=12): G1: no treatment (control group); G2: 9% hydrofluoric acid (HF); G3-6 were separately irradiated with Er:YAG laser using four energy parameters: 2W, 100mj (G3); 3W, 150mj (G4); 4W, 200mj (G5) and 5W, 250mj (G6), respectively; and 20 Hz frequency in long-pulse mode. After silane treatment, a resin composite rod was bonded to each of the porcelain block. The SBS was measured following storage and thermocycling. Data were analyzed by one-way ANOVA, Tamhane and Chi-Square tests.

**Results:**

The highest SBS (12.29±3.04 MPa) was obtained with HF (G2). The lowest SBS (2.23±0.60 MPa) was observed in G4, followed by G3 (1.96±0.76 MPa). G6 had a significantly higher SBS (8.00±2.22 MPa) than other laser irradiation groups.

**Conclusion:**

Although, Er:YAG laser irradiation at 5W, 250mJ/20 Hz was effective in promoting adhesion of resin composite to feldspathic porcelain compared with the control group, it cannot be used as a safe alternative method to HF acid. Laser irradiation with the evaluated parameters in this study does not promote an effective adhesion on porcelain surface to create adequate bond for clinical use.

## Introduction

As the patients’ demand for esthetic dentistry raises, the use of porcelain restorations increases correspondingly. [1-2] Feldspathic porcelain, also known as conventional porcelain, is one of the first dental porcelains used for fabrication of porcelain-fused-to-metal restorations. [3-4] Possessing several favorable mechanical properties, including high flexural strength, high compression resistance and wear resistance, as well as excellent esthetic properties enables this material to imitate the color, reflectivity, and translucency of natural teeth. In addition to biocompatibility and coefficient of thermal expansion similar to tooth structure, it is highly resistant to dissolution in the oral cavity and has low thermal and electrical conductance. [3, 5] However, fracture of porcelain fused-to-metal crowns is a common problem in restorative dentistry. [6-8] Having a prevalence range from 2.3% to 8%, porcelain fractures are reported as the second most common reason for restoration replacement; the first is dental caries. [6]

Fractured porcelains pose an esthetic and functional dilemma both for the patient and the dentist. This problem has raised the demands for development of practical repair options which do not necessitate the removal and remake of the entire restoration. [8] It was *previously believed that* complete removing of prosthesis and constructing a new one result in more acceptable esthetic and durability; [5, 8] but this procedure is demanding, time-consuming, expensive, and also unpleasant for the patients. Therefore, resin composites are strongly advised to be used intra-orally, particularly in less severe cases. [5, 7] Repairing ceramic-based restorations can increase the clinical longevity of failed restorations and offer the dentist and patient a cost-effective alternative option to replacement. [6]

To improve the performance of porcelain repair, porcelain surface-conditioning is required. This procedure increases the surface area and produces micro-porosities on the porcelain surface, causes better adhesion of resin composite, enhances the bond strength, and holds the esthetic at repairing interface for a longer time. Various techniques have been suggested for this strategy particularly air-particle abrasion with aluminum oxide particles; chemical preparation by etching with phosphoric acid, hydrofluoric acid (HF) or acidulated phosphate fluoride (APF) gel; laser irradiation, and combining any of these methods. [9-13]

Using lasers for dental applications has increased rapidly since its invention in 1960. Recently, various types of lasers have been suggested for porcelain surface treatments. To the best of our knowledge, only a few studies with *controversial* results have been carried out regarding the effects of laser as a surface treatment agent on shear bond strength (SBS) of resin composite to fractured porcelain. *Er:YAG laser is one of the* most promising laser types for this purpose. Due to its good interaction with dental structures, *Er:YAG laser *can be a favorable alternative for repair procedures on ceramic materials. [9, 14-15] Nevertheless, the efficacy of this laser on feldspathic porcelain surface is still under debate. [13, 16]

Considering these controversies, in addition to the limitation of related literature, the purpose of this *in vitro* study was to investigate and to compare the effects of Er:YAG laser irradiation with different power output, energy parameters, and hydrofluoric acid (HF) etching on the SBS of a resin composite to feldspathic porcelain. The mode of failure was determined as cohesive, adhesive, or mixed mode. The first null hypothesis claimed no difference between Er:YAG laser irradiation and HF method concerning the mean SBS of resin composite to treated feldspathic porcelain; the second null hypothesis stated that there was no difference among different laser power outputs on the SBS of resin composite to feldspathic porcelain.

## Materials and Method

In this *in vitro* study, 72 experimental blocks were fabricated (10×10×5mm) from feldspathic porcelain (VITA Zahnfabrik H. Rauter GmbH & Co. KG; Bad Säckingen, Germany, A2 Body Shade) in a mold according to the manufacturer’s instruction. The surface of porcelain blocks were then examined using an optical microscope (Olympus Optical Co.; Tokyo, Japan), and the cracked blocks were excluded from the study. Also, the thickness of each specimen was measured with acaliper (Erskine Dental; Marina Del Rey, CA, USA) and the specimens that did not meet the dimensional criteria were excluded.

The surfaces of porcelain blocks were then roughened with a coarse fissure diamond bur (Diatech Dental AG; Heerbrugg, Switzerland) to remove the glaze layer under running water. Thereafter, the specimens were randomly divided into six groups (n=12) according to the type of surface treatment as following:

Group 1(control group): no treatment applied.
Group 2: The samples were etched with 9% HF acid (Porcelain Etch; Ultradent Products Inc., Utah, USA) for 90 seconds, rinsed thoroughly with water spray and then air dried until a frosted appearance was observed.
Groups 3-6: The porcelain surfaces were separately irradiated with Er:YAG laser (Fotona; Ljubljana, Slovenia) with four energy parameters: 2W, 100mj (group 3); 3W, 150mj (group 4); 4W, 200mj (group 5) and 5W, 250mj (group 6). Laser energy was delivered in long pulse mode with a wavelength of 2.94 μm and a repetition rate of 20 Hz on the porcelain surface for 20 seconds under 80% water flow and 40% air flow. The laser optical fiber was placed perpendicular to the porcelain surface at 1mm distance and was moved in a sweeping motion by hand over an area that was 3mm in diameter. To ensure consistent energy density, distance and handpiece angle, the laser handpiece (noncontact R14, Fotona) was attached to a modified surveyor.

Prior to bonding with resin composite, silane agent (Ultradent Products Inc., Utah, USA) was applied on the treated surface of porcelain for one minute, and then air dried. A OptiBond XTR adhesive (Kerr Italia S.p.a.; Salerno, Italy) was then applied using a light brushing motion for 15 seconds, air dried gently for 5 seconds and finally light-cured for 10 seconds by using a light-emitting diode (LED) light curing unit (Coltolux LED; Coltene/Whaledent Inc., OH, USA) with a light intensity of 800 mW/cm [2].

A transparent plastic tube with inner diameter of 3mm and height of 2mm was filled with resin composite (Point 4; Kerr Italia S.p.a., A1 Body Shade) in a one-layer increment technique and was placed perpendicularly at the center of each porcelain block. Subsequently, any excess resin composite was carefully removed from periphery of the tubes with a sharp surgical blade. It was light-polymerized for 20 seconds vertically and 40 seconds circumferentially (each side 20 seconds) to ensure complete polymerization. After the composite buildup, the plastic tube was carefully removed with a scalpel blade, leaving the resin composite rod on the treated adhesive surface of the porcelain block. The specimens were kept moist to avoid drying and cracking during the laboratory procedures. So, all the specimens were stored in distilled water at 37°C and 100% humidity for two weeks and thermocycled 1500 cycles between 5°C to 55°C to simulate the clinic situation with a dwell time of 1-minute in each bath and transfer time of 5 seconds. 

**Figure 1:**
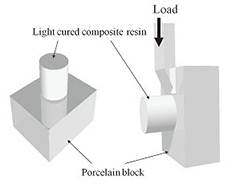
Schematic diagram of shear bond strength measurement (adapted from Chen *et al.*17)

The SBS of resin composite to porcelain specimens was measured using a Universal Testing Machine (*Zwick* GmbH & Co.; Ulm, Germany) at a cross-head speed of 1mm/min until fracture in a compression mode by using a blunt knife-edged apparatus. Shear force was applied to the interface between the composite resin and porcelain block (Figure 1). The maximum load required for debonding the two materials was recorded for each specimen and the SBS was calculated in Mega Pascal (MPa), which is derived by dividing the maximum load force (Newtons) by the bond area (πr²).

After debonding procedure, the surfaces of the specimens were examined by one observer under a stereomicroscope (Olympus Optical Co.; Tokyo, Japan) at 20x magnification to determine the failure mode. The failure mode was classified into three types: adhesive, cohesive or mixed failure and was presented as percentages.

1. Adhesive failure mode was recorded when the resin material rod was completely detached from the porcelain surface.
2. Cohesive failure mode was defined when the bond failure occurred entirely within the resin material, so that the resin material was remained on the porcelain surface.
3. Mixed failure mode was recorded when the bond failure was a combination of the adhesive and cohesive failure modes.

Data were analyzed using SPSS Software (SPSS Inc.; Chicago, IL, U.S.A.), Version 18. Descriptive statistics including the mean, standard deviation, 95% coefficient interval, maximum and minimum of the SBS were evaluated for each group. Normal distribution of the data was confirmed using Kolmogorov-Smirnov test (*p*> 0.05); while homogeneity of variances was not verified using Levene's test (*p*= 0.001). Therefore, one-way analysis of variances (ANOVA) was used for making comparison among all groups and Tamhane’s T2 test for post hoc multiple comparisons were employed. Frequency of the failure modes was analyzed using Chi-Square test. *p*< 0.05 was considered statistically significant.

## Results

Descriptive statistics on the SBS (MPa) of resin composite to porcelain at the fracture for all test groups are tabulated in Table 1. One-way ANOVA revealed the SBS to be considerably different among the study groups (*p*< 0.001). The highest mean SBS (12.29±3.04 MPa) was obtained with HF (group 2) that was significantly higher than the other groups (*p*< 0.05). The lowest mean SBS (1.96±0.76 MPa) was observed in group 3, followed by group 4 (2.23±0.60 MPa).

The results of Tamhane’s T2 test indicated that the SBS of groups 2 and 6 was significantly higher than the control group (*p*< 0.05); but the SBS in groups 3 and 4 was markedly lower than control group (*p*< 0.05). No significant difference was found between group 5 and control group (*p*> 0.05). The results also showed that group 6 (laser irradiation with 5W, 250mj) had a significantly higher mean SBS value than groups 3 (laser irradiation with 2W, 100mj), 4 (laser irradiation with 3W, 150mj) and 5 (laser irradiation with 4W, 200mj) (*p*< 0.05). The results revealed that although group 6 had a significantly higher SBS than groups 3, 4 and 5, and control group, this group had a significantly lower SBS than group 2 (*p*< 0.05) (Table 2).

**Table 1:** Mean shear bond strength (MPa), standard deviation (SD), 95% Coefficient interval and minimum and maximum of resin composite to the feldspathic porcelain (n=12).

**Group**	**Mean±SD**	**95% Confidence Interval**	**Minimum- Maximum**
		**Lower Bound- Upper Bound**	
1	3.76±0.89	3.19-4.32	2.25-4.87
2	12.29±3.04	10.36-14.22	8.09-16.99
3	1.96±0.76	1.47-2.44	0.95-3.22
4	2.23±0.60	1.85-2.62	1.43-3.68
5	2.93±0.47	2.63-3.22	2.22-3.73
6	8.00±2.22	6.59-9.41	5.18-12.03

**Table 2:** Comparison of the mean SBS values between the study groups using Tamhane’s T2 post hoc test (P values)

**Group**	**G1**	**G2**	**G3**	**G4**	**G5**
G2	0.001^†^	-	-	-	-
G3	0.001^†^	0.001^†^	-	-	-
G4	0.001^†^	0.001^†^	0.998^¥^	-	-
G5	0.154^¥^	0.001^†^	0.021^†^	0.069^¥^	-
G6	0.001^†^	0.012^†^	0.001^†^	0.001^†^	0.001^†^

¥: no significant; †: significant

Stereomicroscopic examination of interfacial debonding revealed that the majority of failure modes were adhesive failure followed by mixed failure. Chi-Square test indicated that despite the significant difference observed in the frequency of failure mode in groups 3 and 6 (*p*< 0.05), this result was not obtained in other groups (*p*> 0.05) (Table 3).

**Table 3:** Failure modes of the study groups (n=12)**

**Group**	**Mode of Failure**			***P*** ** Value**
	**Adhesive n** **(%)**	**Cohesive n** **(%)**	**Mixed n** **(%)**	
1	9 (75)	0	3 (25)	0.083^¥^
2	8 (66.7)	0	4 (33.3)	0.284^¥^
3	8 (66.7)	1 (8.3)	3 (25)	0.039^†^
4	7 (58.3)	1 (8.3)	4 (33.3)	0.105^¥^
5	6 (50)	2 (16.7)	4 (33.3)	0.368^¥^
6	11 (91.7)	1 (8.3)	0	0.004^†^

¥: no significant; †: significant

## Discussion

In this study, we experienced that laser irradiation at 5W output power (250mJ/20 Hz) had the highest SBS compared with the other laser surface treatment groups that were treated at a lower output power and also the control group. Also, etching with HF resulted in an even higher SBS than using laser irradiation. This result was in agreement with the results of a study reported by Pedrazzi *et al.* who described deeper conditioning of porcelain that allows a more intense penetration of adhesive system into porcelain and, probably limits the propagation of failure at porcelain. Only Er:YAG laser irradiation with energy of 500mJ/10 Hz and air/water refrigeration showed a well-defined ablation zone. Nevertheless, they concluded that the Er:YAG laser with the mentioned parameters were not effective in promoting the porcelain surface suitable for adhesion, either by ablation of the material or promotion of failure such as cracking due to the photothermal effect. [16]

Laser irradiation creates a rough surface by removing the glass phase of porcelain and increases the micromechanical retention of resin materials. [9, 14-15] However, in the present study, the SBS of resin composite to porcelain after Er:YAG laser treatment was significantly weaker than that of the HF surface treatment method; therefore, the first null hypothesis was rejected. Accordingly, the assumption can be made that laser energy from an Er:YAG laser will not be strongly absorbed in porcelain to create a micro-mechanical retention pattern for more favorable bonding. In agreement with this study, some researchers reported that the Er:YAG laser, even at a higher energy intensity (500mJ), was not able to cause adequate roughness on the porcelain surface and to promote reliable adhesion to the resin composite. [13, 18]

The results of this study indicated that laser irradiation with power output of 4W (200mJ/20 Hz) and the control group produced similar bond strength values. Pedrazzi *et al.* reported that Er:YAG laser with low power energy cannot create consistent changes in the porcelain surface to improve the adhesion. [16] Also, the SBS of the irradiated specimens with power output of 3W (150mJ/20 Hz) and 2W (100mJ/20 Hz) was significantly less than even the control group. Shiu *et al.* also observed that Er:YAG laser irradiating of a feldspathic porcelain surface at 1W output power (100mJ/10 Hz) did not lead to adequate roughening of the surface due to the composition of the porcelain and its reflectance. Therefore, the extent of the superficial changes on the porcelain surface depends on both the energy density of the laser radiation and the type of irradiated porcelain. [13] In this study, a significant difference was also observed among different laser power outputs, thus the second null hypothesis was rejected.

Laser ablation of porcelain surface, produced by low energy density in this study, may obliterate the porosities caused by diamond bur preparation and lead to surface smoothness. Future studies are warranted concerning the effects of low energy output of Er:YAG laser on polishing and smoothing the porcelain. Also, in order to use an Er:YAG laser for superficial treatment of dental porcelains and to achieve a better clinical performance, more studies are still required to examine the different parameters of such laser. Recently, Pedrazzi *et al.* claimed that among various lasers; Er:YAG, Nd:YAG, CO2 and Ti:sapphire, the latter one which is still under clinical trial investigation, was more effective in promoting a favorable surface for adhesion. This laser improved the SBS of porcelain repair as much as using HF did. [16]

In our study, etching with HF showed the highest SBS values compared with other groups, and HF acid was more effective than laser to facilitate better micromechanical retention. This finding was in consistent with previous reports. [9, 13-14, 16, 18] Akyil *et al.* concluded that HF acid etching is the most effective surface treatment for increasing the shear bond strength between a repair composite resin and a feldspathic ceramic surface. The shear bond strength after laser irradiation can be increased by HF acid etching, but the strength of the bond is still smaller than that after HFA etching alone. [19] Etching with HF acid can produce a proper surface texture, roughness and irregularity by dissolving the crystalline and glassy phases of the porcelain surface. [18, 20-21] However, there are studies that claim the therapeutic effects of HF are not significantly better than the other treatments. [20-21] Furthermore, intraoral use of HF acid is well recognized for having hazardous effects on soft tissues, so it is not a practical method in dentistry, particularly if used for intraoral porcelain repairs. [4, 8, 13]

We also showed that the differences in frequencies of the failure mode were not significant within control and test groups except for the groups that were irradiated with 2W (100 mJ/20 Hz) and 5W (250 mJ/20 Hz) power output. Based on an *analysis of the failure sites,*the major failure pattern was the adhesive failure. According to these findings, no cohesive failure was observed in the porcelain and only a few samples had a cohesive failure in the resin composite. It may be inferred that in spite of improvement of the bond strength between the resin composite and the feldspathic porcelain surface through various surface treatments, this bond was still not strong enough and was lower than the cohesive resistance of resin composite and the porcelain.

As strongly recommended by relevant researches, [20, 22-23] silane coupling agent was used in this study to improve bonding between the resin composite and porcelain by producing chemical adhesion. This study had limitations in *simulating* real *clinical* situations; thus future studies are required to use an experimental model with conditions that more closely resembles the *in-vivo* environment.

## Conclusion

Considering the limitations of the present *in vitro* study, the following conclusions could be drawn:

1. HF acid created a higher SBS than the Er:YAG laser irradiation with different power output energies (*p*< 0.05); however, HF was the most effective surface treatment and produced the strongest bonds.
2. The Er:YAG laser irradiation at 5W, 250 mJ/20 Hz was effective in promoting adhesion of resin composite to feldspathic porcelain surface than lower power output energies. Nevertheless, it cannot be used as a safe alternative method compared to HF acid based on the parameters evaluated in this study.
